# Distinct Effects of EGFR Ligands on Human Mammary Epithelial Cell Differentiation

**DOI:** 10.1371/journal.pone.0075907

**Published:** 2013-10-04

**Authors:** Chandrani Mukhopadhyay, Xiangshan Zhao, Dulce Maroni, Vimla Band, Mayumi Naramura

**Affiliations:** 1 Eppley Institute for Research in Cancer and Allied Diseases, University of Nebraska Medical Center, Omaha, Nebraska, United States of America; 2 Department of Genetics, Cell Biology and Anatomy, College of Medicine, University of Nebraska Medical Center, Omaha, Nebraska, United States of America; University of Central Florida, United States of America

## Abstract

Based on gene expression patterns, breast cancers can be divided into subtypes that closely resemble various developmental stages of normal mammary epithelial cells (MECs). Thus, understanding molecular mechanisms of MEC development is expected to provide critical insights into initiation and progression of breast cancer. Epidermal growth factor receptor (EGFR) and its ligands play essential roles in normal and pathological mammary gland. Signals through EGFR is required for normal mammary gland development. Ligands for EGFR are over-expressed in a significant proportion of breast cancers, and elevated expression of EGFR is associated with poorer clinical outcome. In the present study, we examined the effect of signals through EGFR on MEC differentiation using the human telomerase reverse transcriptase (hTERT)-immortalized human stem/progenitor MECs which express cytokeratin 5 but lack cytokeratin 19 (K5^+^K19^-^ hMECs). As reported previously, these cells can be induced to differentiate into luminal and myoepithelial cells under appropriate culture conditions. K5^+^K19^-^ hMECs acquired distinct cell fates in response to EGFR ligands epidermal growth factor (EGF), amphiregulin (AREG) and transforming growth factor alpha (TGFα) in differentiation-promoting MEGM medium. Specifically, presence of EGF during *in vitro* differentiation supported development into both luminal and myoepithelial lineages, whereas cells differentiated only towards luminal lineage when EGF was replaced with AREG. In contrast, substitution with TGFα led to differentiation only into myoepithelial lineage. Chemical inhibition of the MEK-Erk pathway, but not the phosphatidylinositol 3-kinase (PI3K)-AKT pathway, interfered with K5^+^K19^-^ hMEC differentiation. The present data validate the utility of the K5^+^K19^-^ hMEC cells for modeling key features of human MEC differentiation. This system should be useful in studying molecular/biochemical mechanisms of human MEC differentiation.

## Introduction

Molecular profiling of breast cancer revealed unexpected heterogeneity of this disease [1,2]. According to these studies, breast cancers can be categorized into several separate subtypes which share considerable similarities with various developmental stages of normal mammary epithelial cells (MECs). Consequently, a hypothesis was proposed that individual types of cancer might arise from malignant transformation of comparable normal MECs [3]; however, more recent studies employing lineage tracing [4–6], marker analysis [7], transplantation [8] and other techniques [7,9,10] began to uncover the complexity and the plasticity of the normal and pathological mammary epithelial developmental processes.

One of the difficulties of studying detailed molecular/biochemical mechanisms of normal and pathological MEC differentiation is the lack of accessible models. Sources of normal human MECs include reduction mammoplasty specimen and normal margins of surgically-excised tumor tissues, but primary cells have limited lifespan *in vitro*, and are not always readily available. Underlying genetic, epigenetic and environmental variations between donors may also be a concern. Genetically-engineered mouse models provide powerful tools to address important biological questions [11], but due to inherent differences between human and mouse mammary gland physiology, observations in mice may not directly translate to human pathophysiology. There are also technical challenges to follow developmental processes as they progress *in vivo*. To address these issues, Band and colleagues have previously established human telomerase reverse transcriptase (hTERT)-immortalized MEC lines that can be propagated indefinitely in a stem/progenitor-like undifferentiated state but can be induced to differentiate into luminal as well as myoepithelial lineages *in vitro* under defined conditions [12]. Because they are amenable to complex manipulations, these models should be useful for studying mechanisms of MEC self-renewal, differentiation, transformation and cancer progression.

In the present study, we sought to characterize and validate this cell line model further by focusing on the effects of signals through epidermal growth factor receptor (EGFR) on *in vitro* differentiation. EGFR is recognized as a critical regulator of mammary gland development [13]. A naturally-occurring mutation in the *egfr* gene in mice (*wa-2*) has been known to impair lactation [14]. On the ligand side, although multiple ligands are known to bind to EGFR, only amphiregulin (AREG)-deficient mice showed significant defects in the development of mammary gland, suggesting redundant as well as unique functions of individual EGFR ligands [15]. Links between the EGFR-dependent processes and mammary gland pathophysiology are further reinforced by the observations that EGFR is either amplified or overexpressed in a considerable proportion of basal-like breast cancers [16,17]; transcripts for EGFR ligands such as epidermal growth factor (EGF), AREG and transforming growth factor alpha (TGFα) are frequently upregulated in human breast cancer samples and a majority of breast cancers that express high levels of TGFα also co-express EGFR, suggesting a potential autocrine loop [18–22]. All these point towards the crucial roles of EGFR and its ligands in the biology of the mammary gland and breast cancer. As for the biochemical mechanisms, it was previously reported that sustained activation of the EGFR-MEK-Erk pathway was required for myoepithelial differentiation of primary human MECs [23]. Taking these prior observations into consideration, here we examined the effects of three EGFR ligands, EGF, AREG and TGFα in the differentiation-promoting MEGM medium on the hTERT-immortalized stem/progenitor hMEC line characterized by the presence of cytokeratin 5 and absence of cytokeratin 19 (K5^+^K19^-^ hMEC).

## Materials and Methods

### Cell culture

The development and initial characterizations of hTERT-immortalized stem/progenitor hMEC line defined by the presence of K5 and absence of K19 (K5^+^K19^-^ hMECs) were reported previously [12]. Cells were routinely maintained in the DFCI-1 medium [24]. All experiments were performed within 20 passages in culture.

For *in vitro* differentiation, K5^+^K19^-^ hMEC cells were cultured in MEGM medium (MEBM, (Lonza, Walkersville, MD, USA) supplemented with B27 (Life Technologies, Carlsbad, CA, USA), 4 µg/mL heparin (Sigma-Aldrich, St. Louis, MO, USA), 5 µg/mL insulin (Sigma-Aldrich), 30 ng/mL (5 nM) EGF (Life Technologies), 20 ng/mL FGF (Life Technologies) and 0.5 µg/mL hydrocortisone (Sigma-Aldrich)). EGF was replaced with AREG or TGFα (5 nM unless specified otherwise) where indicated. Cells were passaged once a week and seeded at 2 × 10^5^ in a 60 mm dish (for flow cytometry analysis) or 2 × 10^4^ cells/well on the top of the 12 mm glass coverslip in the 24-well plate (for confocal imaging analysis).

### Antibodies

Antibodies used for this study are listed in Table 1.

**Table 1:** List of antibodies used for this study.

	Vendor	Clone Name	Catalog Number
Phosphotyrosine	EMD Millipore	4G10	05-321
EGFR	Santa Cruz Biotechnology	Rabbit polyclonal	sc-03
Phospho-p44/42 MAPK (Erk1/2) (Thr202/Tyr204)	Cell Signaling Technology	Rabbit polyclonal	9101
p44/42 MAPK (Erk1/2)	Cell Signaling Technology	Rabbit polyclonal	9102
Phospho-Akt (Ser473)	Cell Signaling Technology	D9E	4060
Akt	Cell Signaling Technology	C67E7	4691
HSC70	Santa Cruz Biotechnology	B-6	sc-7298
MUC1	BD Biosciences	HMPV	550486
MUC1-FITC	BD Biosciences	HMPV	559774
EpCAM-APC	BD Biosciences	EBA-1	347200
CD49f-PE-Cy7	eBioscience	eBioGoH3	25-0495
CD10-APC eFluor 780	eBioscience	SN5c	8047-0108
Cytokeratin 5	Covance	Rabbit polyclonal	PRB-160P
Cytokeratin 5/6-FITC	EMD Millipore	D5/16B	FCMAB291F
Alpha Smooth Muscle Actin	Sigma-Aldrich	1A4	A2547
HRP-Protein A	Invitrogen	N/A	10-1023
HRP-Rabbit anti-Mouse IgG (H+L) conjugate	Invitrogen	Rabbit polyclonal	R21455
Alexa Fluor 488 Donkey anti-Rabbit IgG (H+L)	Invitrogen	Donkey polyclonal	A-21206
Alexa Fluor 594 Donkey anti-Mouse IgG (H+L)	Invitrogen	Donkey polyclonal	A-21203

### Immunofluorescence and confocal microscope image analysis

Immunofluorescence and confocal image analyses were performed as described previously [12]. Briefly, cells were grown on 12 mm glass coverslips, fixed with 4% paraformaldehyde in phosphate buffered saline (PBS) and permeabilized with 0.5% Triton X-100 for 5 minutes. After blocking non-specific binding sites with 5% goat serum for 1 hour, samples were incubated with the primary anti-K5 (1:2000) and anti-MUC1 (1:500) antibodies in 1% goat serum-containing PBS overnight at 4°C. After three washes with PBS, anti-rabbit IgG Alexa Fluor 488 and anti-mouse IgG Alexa Fluor 633 were added at 1:1000 dilutions in 1% goat serum containing PBS and incubated at room temperature for 1 hour. After washes in PBS and in water, nuclei were visualized with DAPI by mounting with VECTASHIELD Hard Set mounting medium (Vector Laboratories, Burlingame, CA). Images were captured with a Zeiss LSM 710 META laser scanning confocal microscope (Carl Zeiss Microscopy GmbH, Jena, Germany).

### Flow Cytometry

Cells were detached from culture plates by trypsin, filtered through a 40 µm nylon mesh (BD Biosciences) to ensure single cellularity, re-suspended in ice-cold FACS buffer (PBS/1% bovine serum albumin) at 10^6^ cells/200 µl and incubated for 20 minutes with antibodies against EpCAM, CD10, MUC1 and CD49f. To stain intercellular K5, cells were first surface-stained with anti-EpCAM and anti-CD49f antibodies, fixed with 4% paraformaldehyde at room temperature for 15 minutes, washed once with FACS buffer and once with FACS buffer containing 0.5% saponin, and then incubated with anti-K5 antibody (1:100) in FACS buffer with saponin at room temperature for 30 minutes. Cells were then washed once in FACS buffer with saponin, once in FACS buffer before proceeding for flow cytometry analysis. Data were acquired on an LSRII (BD Biosciences) and analyzed using FlowJo software (Tree Star Inc., Ashland, OR, USA).

### Immunoblotting assays

Cells were grown to 70-80% confluence in DFCI-I medium and starved of serum/growth factors in D3 medium [25] for 48 hours. For stimulation with EGFR ligands, starved cells were either left untreated or treated with 5 nM AREG, 5 nM EGF or 5 nM TGFα. After indicated stimulation period, cells were lysed in cold lysis buffer (0.5% Triton X-100, 50 mM Tris-HCl (pH 7.5), 150 mM sodium chloride, 1 mM phenylmethylsulfonyl fluoride, 1 mM sodium orthovanadate, and 10 mM sodium fluoride). Cell lysates were centrifuged at 10,000 rpm at 4 °C for 30 min and protein concentrations in the supernatant was quantified using Bradford reagent (Bio-Rad, Richmond, CA, USA). Cell lysates (20 µg protein equivalent) were resolved by SDS-PAGE, transferred to polyvinylidene difluoride membranes (PerkinElmer Life Sciences, Waltham, MA, USA) and incubated with primary antibodies against pY (4G10), EGFR, pErk1/2, Erk1/2, pAkt, Akt or HSC70 followed by incubation with HRP-conjugated protein A (for rabbit antibodies) or rabbit anti-mouse antibody (for mouse monoclonal antibodies). Primary antibodies were diluted to 1:1000 in TBS-T (20 mM Tris-HCl (pH 8.0), 150 mM sodium chloride, and 0.1% v/v Tween-20), except anti-pY (4G10) which was used at 1:4000. Secondary antibodies were diluted to 1:25,000 in TBS-T. The enhanced chemiluminescence signals were recorded using a light-sensitive film (GeneMate Blue Lite Autorad Film).

### Thymidine Incorporation Assay

Proliferation assay was performed essentially as described previously [26]. K5^+^K19^-^ hMEC cells were seeded in 24-well plates at 2 × 10^4^/well in DFCI-1 medium. Next day, cells were rinsed and serum/growth factor-starved in D3 medium for 24 hours. Cells were then left unstimulated or stimulated with AREG, EGF or TGFα (all ligands at 5 nM) for 48 hours. [^3^H] thymidine (4 µCi/ml) was added for the last 6 hours of incubation. To terminate incubations, unincorporated radioactivity was removed by washing cells once with ice-cold PBS followed by the addition of 10% trichloroacetic acid for 30 minutes at 4°C. Next, wells were washed with ice-cold PBS and solubilized with 0.2 M NaOH at room temperature. The radioactivity was determined by liquid scintillation counting.

### Statistical Analysis

Statistical analysis was performed using GraphPad Prism package (GraphPad Software, La Jolla, CA, USA).

## Results

### Characterization of *in vitro* differentiated K5^+^K19^-^ hMEC cells by flow cytometry

K5^+^K19^-^ hMEC cells maintain undifferentiated morphology and marker expression in DFCI-1 medium but they can be induced to differentiate towards both luminal and myoepithelial lineages when cultured in MEGM medium [12]. Original studies were carried out using immunofluorescence-based analyses. While this method is well-suited to correlate cell morphology, marker expression and its localization, objective quantitative assessment of individual markers require alternative approaches. Therefore, we sought to examine the differentiation process more quantitatively by flow cytometry.

**Figure 1:**
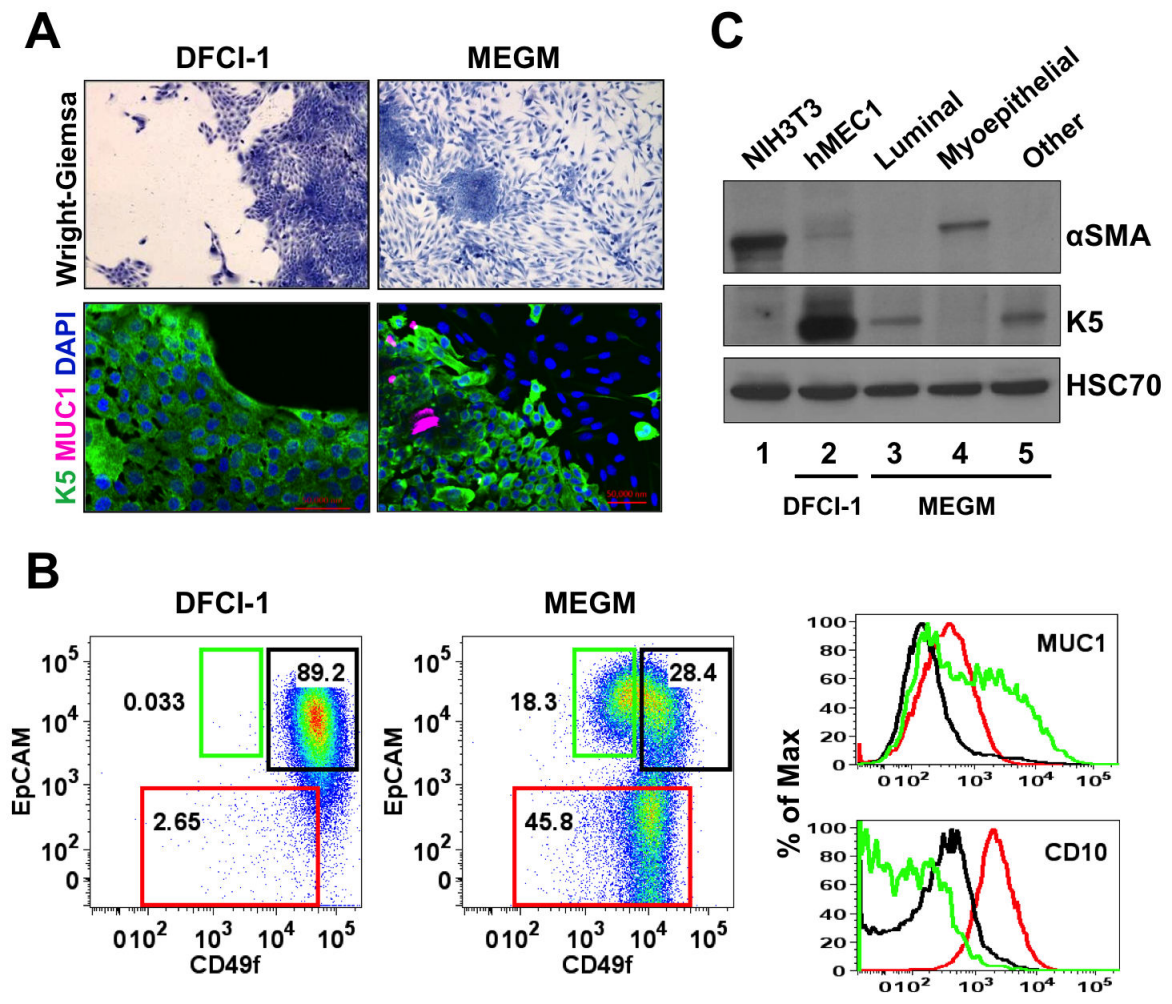
In vitro differentiation of K5^+^K19^-^ h*TERT*-immortalized mammary epithelial cells. Cells were either maintained under non-differentiating condition (DFCI-1 medium) or propagated under differentiation-promoting condition (MEGM medium containing 5 nM EGF) for three weeks and cell morphology and marker expressions were evaluated. Shown are representative results from more than 10 independent experiments with similar outcome. (**A**) Overall cell morphology was assessed by Wright-Giemsa staining (top panels) and K5 (green) and MUC1 (purple) expression was assessed by immunofluorescence microscopy (bottom panels). Nuclei were visualized with DAPI (blue). Red bars indicate 50 µM. (**B**) Expression of CD49f, EpCAM, MUC1 and CD10 was assessed by flow cytometry. Gates for CD49f^lo^EpCAM^hi^ (luminal, green box), EpCAM^lo^ (myoepithelial, red box) and CD49f^hi^EpCAM^hi^ (undifferentiated, black box) cells are indicated. Histograms on the right indicate levels of MUC1 (luminal marker, top) and CD10 (myoepithelial marker, bottom) in cells propagated in MEGM medium. Green lines represent the levels of MUC1 (top) or CD10 (bottom) in the CD49f^lo^EpCAM^hi^ (luminal) population, red lines are for the EpCAM^lo^ (myoepithelial) population and black lines for the CD49f^hi^EpCAM^hi^ (undifferentiated) population. (**C**) Expression of α-smooth muscle actin (αSMA) and K5 was assessed by immunoblotting. NIH3T3 cells (lane 1) were included as a positive control for αSMA. Lane 2: K5^+^K19^-^ hMECs maintained in DFCI-1 medium; Lanes 3-5: K5^+^K19^-^ hMECs were differentiated in MEGM medium and sorted into CD49f^lo^EpCAM^hi^ (luminal), EpCAM^lo^ (myoepithelial) and CD49f^hi^EpCAM^hi^ (undifferentiated) populations. Membrane was probed for HSC70 to ensure equal loading.

To this end, K5^+^K19^-^ hMEC cells were cultured for three weeks in MEGM medium containing 5 nM EGF (see Materials and Methods for the detailed composition of this medium) and cell differentiation was examined by morphology, immunofluorescence and flow cytometry. In the DFCI-1 medium, all K5^+^K19^-^ hMEC cells maintained tightly-packed epithelial morphology (Figure 1A). In line with previous reports, all cells maintained in the DFCI-1 medium expressed K5 and lacked MUC1 (Figure 1A). When analyzed by flow cytometry, most cells expressed intermediate to high levels of epithelial cell adhesion molecule (EpCAM) and high levels of integrin α6 (CD49f) (Figure 1B). On the other hand, when cultured in MEGM medium for three weeks, cells organized themselves into two distinct populations, one characterized by the tightly-packed epithelial morphology and the other spindle-shaped cells surrounding the tight epithelial colonies (Figure 1A). Spindle-shaped cells migrated away from the packed epithelial colonies even at low cell confluence while cells with epithelial morphology remained in the colonies even at high cell confluence. Changes in cell morphology were not the direct consequence of culture confluence. Immunofluorescence imaging revealed that spindle-shaped cells lost expression of K5 whereas a fraction of cells within the tightly-packed epithelial colonies expressed MUC1. MUC1^pos^ cells were found mostly in the center of the colonies. All these data are consistent with previous findings [12]. Flow cytometry analyses identified two distinct populations based on EpCAM expression (EpCAM^hi^ and EpCAM^lo^) and EpCAM^hi^ cells could be further separated based on CD49f expression (CD49f^hi^EpCAM^hi^ and CD49f^lo^EpCAM^hi^). Previous studies on normal primary hMECs reported that CD49f was expressed highly in stem/early progenitor cells while EpCAM was a marker for luminal lineage [27–30]. Therefore, we reasoned that EpCAM^hi^ population might be luminal cells that form tightly-packed colonies while EpCAM^lo^ population are spindle-shaped myoepithelial cells. To confirm this, we evaluated the expression of MUC1, a luminal marker, and CD10, a myoepithelial marker, in individual populations. As shown in Figure 1B, MUC1 was most highly expressed in the CD49f^lo^EpCAM^hi^ population whereas CD10 expression was the highest in EpCAM^lo^ cells. To further establish the identity of each population, we sorted these three populations (Figure 1C) and subjected them to immunoblotting analysis for known markers of epithelial differentiation. The EpCAM^lo^ population lost K5 expression but up-regulated the expression of α-smooth muscle actin, a widely-accepted marker of myoepithelial differentiation. Changes in K5 expression was also confirmed by flow cytometry analysis (Figure S1). Altogether, we conclude that the tightly-packed epithelial colonies contain CD49f^hi^EpCAM^hi^ and CD49f^lo^EpCAM^hi^ cells, CD49f^lo^EpCAM^hi^ population contains MUC1^pos^ cells, i.e., more differentiated luminal cells than CD49f^hi^EpCAM^hi^ cells, and the surrounding spindle-shaped cells correspond to the EpCAM^lo^ population.

### Effects of various EGFR ligands on K5^+^K19^-^ hMEC differentiation

Signals through EGFR play critical roles in mammary gland development and homeostasis. Seven different ligands have been identified for EGFR; EGF, amphiregulin (AREG), transforming growth factor alpha (TGFα), heparin-binding EGF-like growth factor (HB-EGF), betacelluin, epiregulin and epigen. They are known to differ in their binding affinity to EGFR, activate distinct biochemical pathways and have diverse effects on receptor trafficking, degradation and recycling [31,32]. Therefore, we sought to compare the effects of two other EGFR ligands relevant to mammary gland development and breast cancer, AREG and TGFα, on K5^+^K19^-^ hMEC cell differentiation.

**Figure 2:**
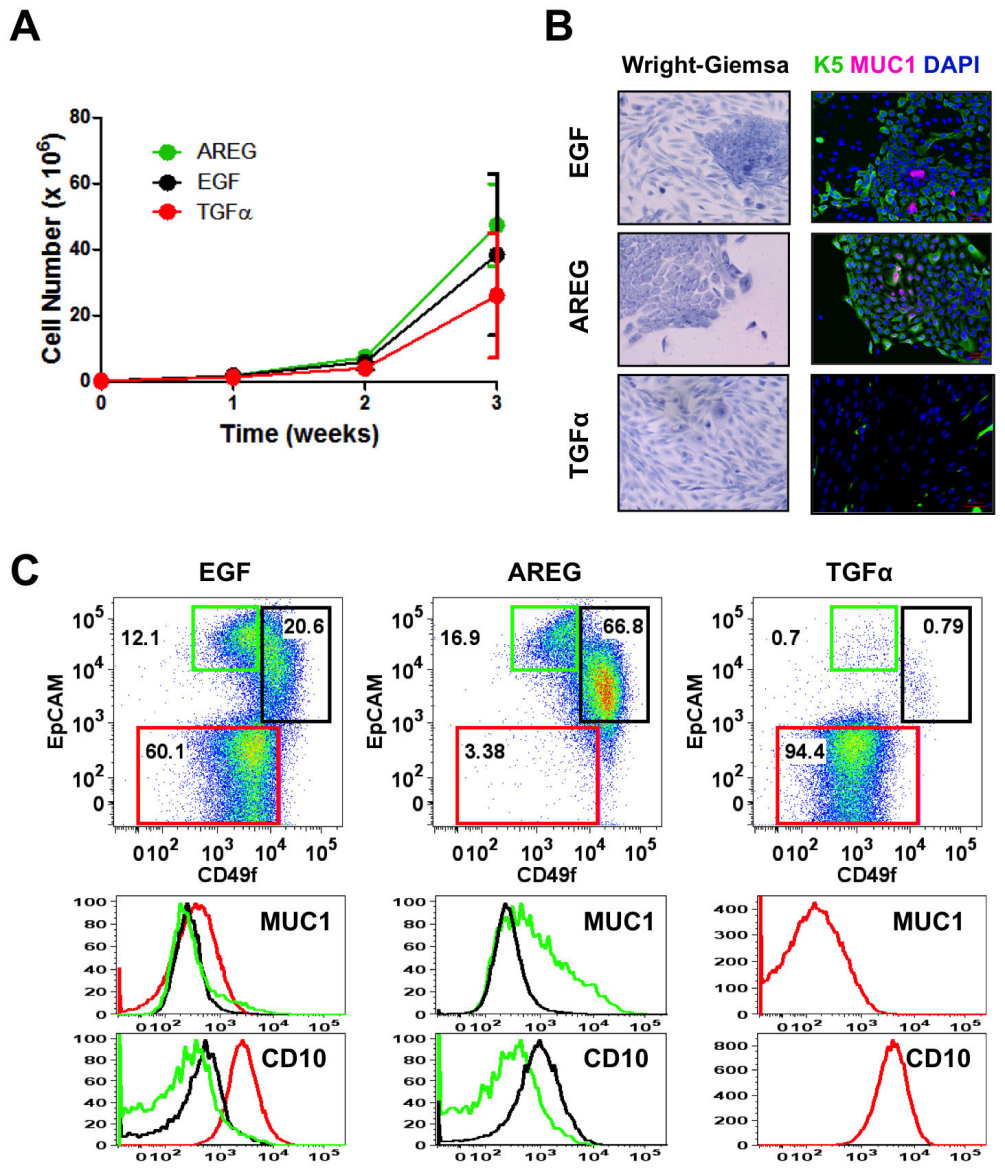
Effects of various EGFR ligands on K5^+^K19^-^ hMEC differentiation in MEGM medium. Cells were propagated in modified MEGM media where EGF was substituted with either AREG or TGFα and morphology and marker expressions were analyzed after three weeks. (**A**) Cell growth during differentiation. Two hundred thousand (2 x 10^5^) K5^+^K19^-^ hMECs were seeded in 60 mm dishes in modified MEGM media with indicated EGFR ligands. Cell numbers were determined every week. Shown are averages from 4 independent experiments. Error bars indicate standard errors. There was no statistically significant difference between groups in cell number at each time point by one-way ANOVA with Bonferroni multiple comparisons. (**B**) Overall cell morphology was assessed by Wright-Giemsa staining (left panels) and K5 (green) and MUC1 (purple) expression was assessed by immunofluorescence microscopy (right panels). Nuclei were visualized with DAPI (blue). Red bars indicate 50 µM. (**C**) Expression of CD49f, EpCAM, MUC1 and CD10 was analyzed by flow cytometry. Gates and percentages for CD49f^lo^EpCAM^hi^ (luminal, green box), EpCAM^lo^ (myoepithelial, red box) and CD49f^hi^EpCAM^hi^ (undifferentiated, black box) populations are indicated in the top panels. Middle and bottom panels are histograms for MUC1 (middle) and CD10 (bottom). Green lines represent the levels of MUC1 (middle) or CD10 (bottom) in the CD49f^lo^EpCAM^hi^ (luminal) population, red lines are for the EpCAM^lo^ (myoepithelial) population and black lines for the CD49f^hi^EpCAM^hi^ (undifferentiated) population. Histograms for EpCAM^lo^ (myoepithelial) populations in AREG-treated cells and CD49f^lo^EpCAM^hi^ (luminal) and CD49f^hi^EpCAM^hi^ (undifferentiated) populations in TGFα-treated are not shown in the overlays due to extremely small cell numbers. Though the difference in MUC1 expression between EGF-treated cell populations was not as robust as in Figure 1B in this particular experiment, MUC1 mean fluorescence intensity for CD49f^lo^EpCAM^hi^ (luminal) cells (642) was higher than that of EpCAM^lo^ (myoepithelial) or CD49f^hi^EpCAM^hi^ (undifferentiated) cells (437 and 337, respectively). (**B**) and (**C**) are representative results from 6 independent experiments with similar outcome.

To this end, K5^+^K19^-^ hMEC cells were cultured in modified MEGM medium where EGF was substituted with the same concentrations (5 nM) of AREG or TGFα. We selected this concentration because cells did not differentiate at lower concentrations of AREG (Figure S2). As shown in Figure 2A, there was no significant difference in cell growth when cells were maintained in MEGM medium containing AREG or TGFα from those cultured in the same medium containing EGF. However, cell acquired considerably different morphology under these conditions; specifically, almost all K5^+^K19^-^ hMEC cells cultured in the presence of AREG formed tight epithelial colonies, and very few spindle-shaped cells were observed. In contrast, a majority of cells cultured in the presence of TGFα turned spindle-shape. These contrasting morphologies are reflected in the expression patterns of K5, MUC1, EpCAM, CD49f and CD10. AREG-treated cells maintained the expression of K5 and those in the middle of the colonies expressed MUC1 whereas a considerable fraction of TGFα-treated cells lost K5 expression and no MUC1-expressing cells were observed (Figure 2B). Flow cytometry analysis revealed the presence of a significant proportion of CD49f^lo^EpCAM^hi^ (luminal) cells and very few EpCAM^lo^ (myoepithelial) cells when cultured with AREG, while most cells were EpCAM^lo^ (myoepithelial) when cultured in the presence of TGFα (Figure 2C). CD49f^lo^EpCAM^hi^ (luminal) cells emerged in the presence of AREG were essentially indistinguishable from those emerged in the presence of EGF in that they maintained expression of K5 (as seen by immunofluorescence) and upregulated MUC1. Likewise, EpCAM^lo^ (myoepithelial) cells emerged in the presence of TGFα shared all the key characteristics with EGF-induced counterparts, i.e., they lost K5 expression and upregulated CD10. Altogether, we concluded that AREG-supplemented MEGM medium promoted the emergence of luminal cells whereas TGFα favored myoepithelial cells.

**Figure 3:**
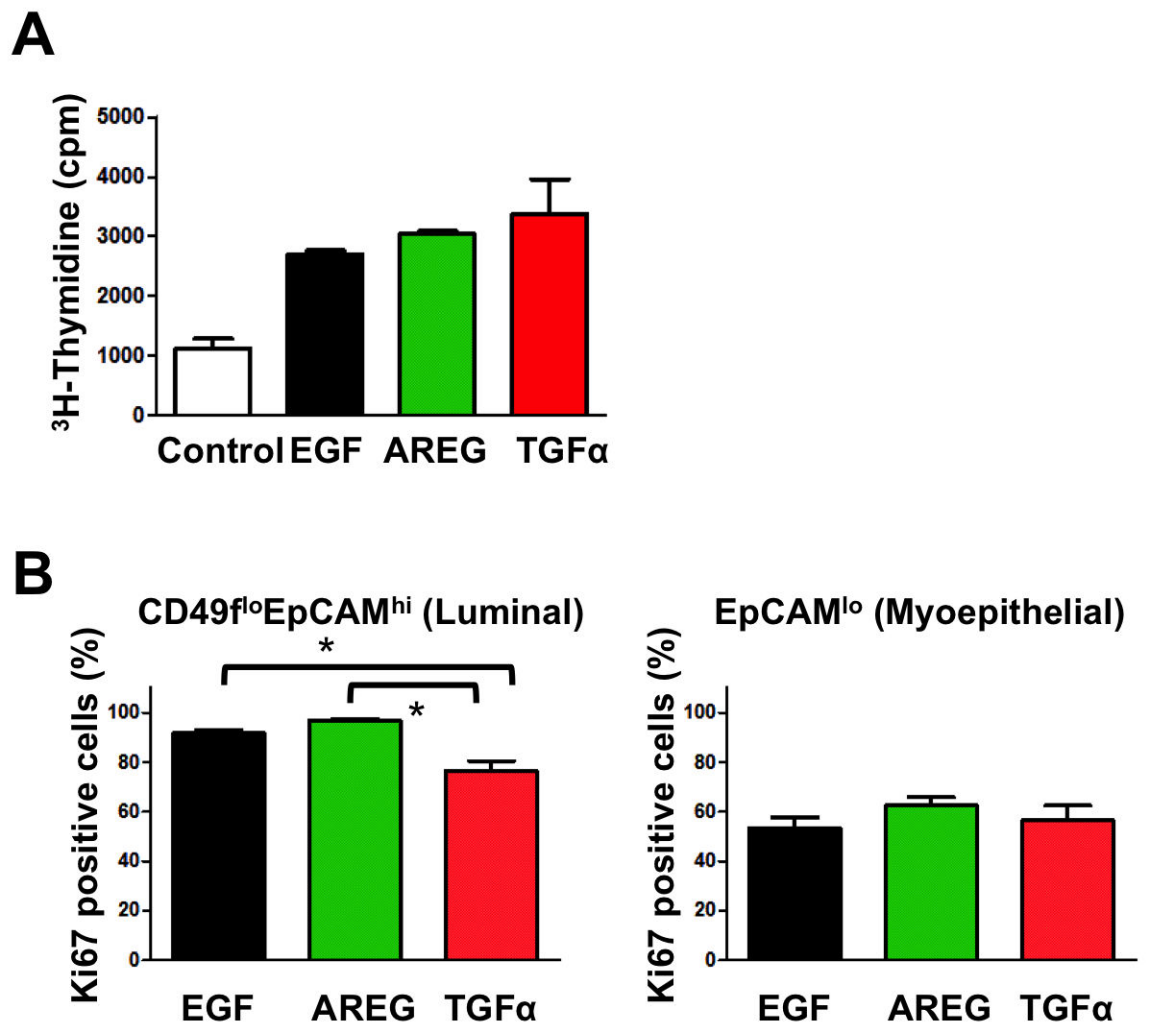
All EGFR ligands support growth of K5^+^K19^-^ hMECs before and after differentiation. (**A**) Undifferentiated K5^+^K19^-^ hMECs were starved of serum/growth factors for 24 hours in D3 medium before being left unstimulated (Control) or stimulated with AREG, EGF or TGFα (all at 5 nM) for 24 hours. Cell growth was assessed by [^3^H] thymidine incorporation for the last 6 hours of incubation. A representative result from 2 independent experiments run in triplicates is shown. Error bars indicate standard errors. (**B**) K5^+^K19^-^ hMECs were propagated in MEGM medium (with EGF) to induce differentiation. Differentiated luminal (CD49f^lo^EpCAM^hi^) and myoepithelial (EpCAM^lo^) cells were separated by FACS and plated in modified MEGM medium containing either AREG, EGF or TGFα (all at 5 nM). The percentage of proliferating cells was assessed by the expression of Ki67. Each condition was run in 5 replicates. Error bars represent standard errors. The difference between EGF and TGFα, as well as that between AREG and TGFα in CD49f^lo^EpCAM^hi^ cells was statistically significant at p<0.05 when analyzed by one-way ANOVA with Bonferroni multiple comparisons.

These results may indicate that different EGFR ligands, when administered as a part of MEGM medium, can directly regulate the lineage specification of K5^+^K19^-^ hMEC cells. Alternatively, it is also conceivable that K5^+^K19^-^ hMEC cells follow an intrinsic differentiation program and each EGFR ligand promotes the survival and/or proliferation of distinct populations. According to the latter hypothesis, AREG should preferentially promote the survival and/or proliferation of luminal cells, TGFα should support only myoepithelial cells while EGF should function both on luminal as well as myoepithelial cells. To test this hypothesis, we first evaluated the effects of various EGFR ligands on K5^+^K19^-^ hMEC short-term cell growth. When undifferentiated K5^+^K19^-^ hMEC cells were starved of serum/growth factors in D3 medium [25] and re-stimulated with EGF, AREG or TGFα, we did not detect significant difference in cell growth (Figure 3A). We next considered the possibility that each EGFR ligand may show preferential effects only on differentiated cells. To test this, we first allowed K5^+^K19^-^ hMECs to differentiate *in vitro* in MEGM medium (containing EGF), sorted CD49f^lo^EpCAM^hi^ (luminal) and EpCAM^lo^ (myoepithelial) populations by flow cytometry and cultured them in modified MEGM medium containing EGF, AREG or TGFα for 72 hours. Sorted cells maintained their morphology during this treatment (Figure S3). When cell proliferation was examined by the expression of the proliferation marker Ki67, the percentages of Ki67^pos^ (proliferating) cells were slightly reduced in CD49f^lo^EpCAM^hi^ (luminal) cells treated with TGFα. Nevertheless, all three EGFR ligands supported proliferation of sorted cells (Figure 3B). Importantly, no significant cell death was observed either morphologically or by Annexin V staining throughout the course of differentiation in all three conditions (data not shown).

Combined with data that there was no significant difference in cell growth during three week differentiation (Figure 2A), these data collectively demonstrate that EGF, AREG and TGFα in MEGM medium show little difference in supporting growth and survival of K5^+^K19^-^ hMEC cells either before or after *in vitro* differentiation, suggesting that the observed differences in K5^+^K19^-^ hMEC differentiation in MEGM medium is not due to preferential survival and expansion of one population, but that EGFR ligands are likely to function directly on hMEC lineage specification in the context of this culture medium.

### Biochemical consequences of EGFR engagement

**Figure 4:**
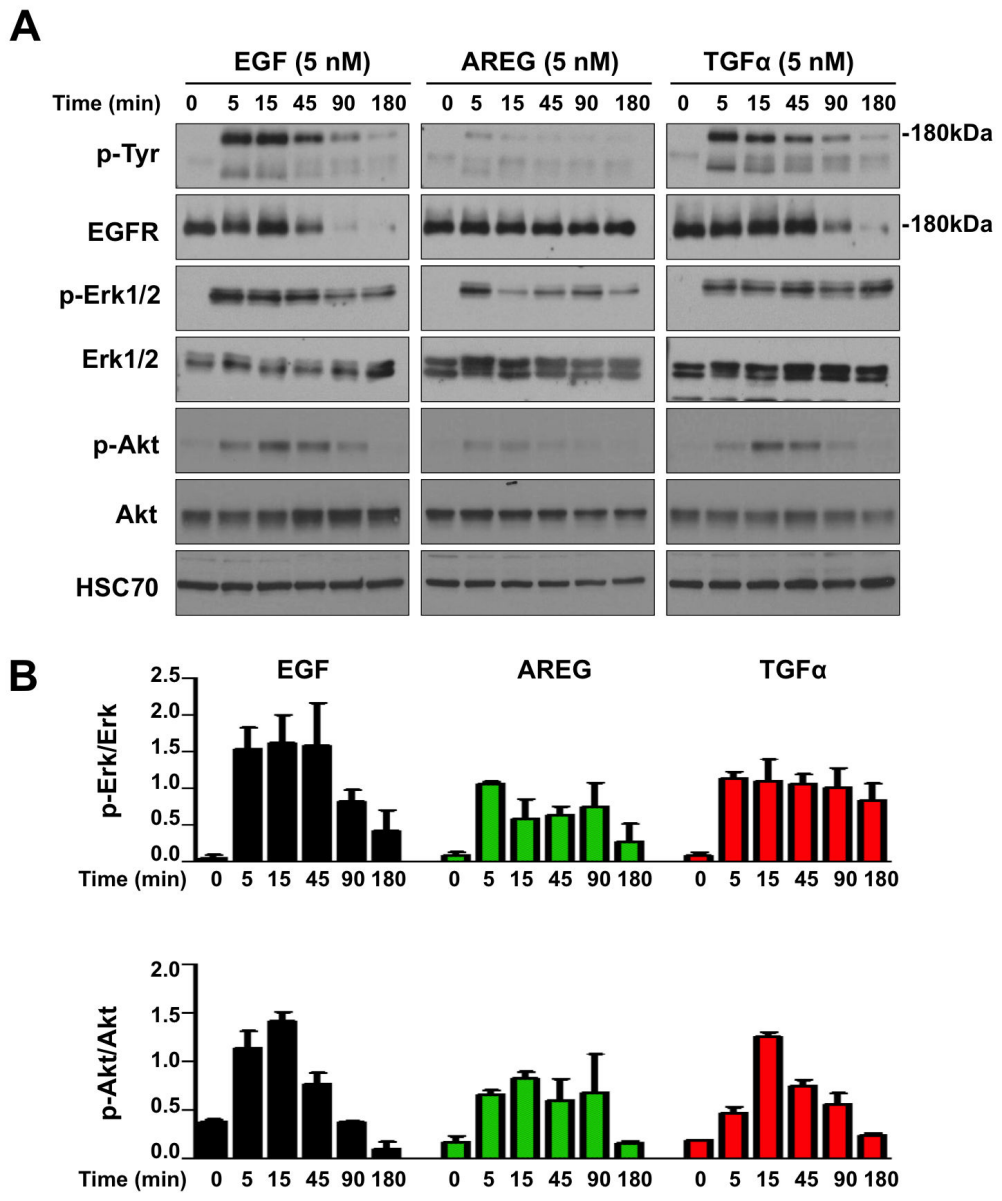
Biochemical consequences of EGFR engagement with various ligands. Undifferentiated K5^+^K19^-^ hMECs were starved of serum/growth factors for 48 hours in D3 medium before being stimulated with 5 nM EGF, AREG or TGFα for indicated period. Cell lysate was analyzed by immunoblotting. (**A**) Immunoblot results of phosphotyrosine (p-Tyr), total EGFR, phospho-p44/42 MAPK (p-Erk1/2), total p44/42 MAPK (Erk1/2), phospho-Akt (p-Akt) and total Akt. HSC70 was used as loading control. A representative of 2 independent experiments is shown. (**B**) Results from 2 independent experiments were quantitated by densitometry and ratios of p-Erk/total Erk and p-Akt/total Akt were plotted. Shown are averages of 2 experiments; error bars indicate standard errors. Y axis is in arbitrary unit.

To begin to dissect the mechanisms how different EGFR ligands regulate K5^+^K19^-^ hMEC cell fate, we first investigated short-term biochemical changes upon receptor engagement. In the present study, we focused on two major biochemical pathways downstream of EGFR, MEK-Erk and phosphatidylinositol 3-kinase (PI3K)-Akt pathways. K5^+^K19^-^ hMEC cells maintained in the DFCI-1 medium were starved for 48 hours in D3 medium. Cells were then stimulated with 5 nM of AREG, EGF or TGFα for up to 180 minutes (Figure 4). Consistent with the published literature [31,32], AREG induced little phosphorylation on EGFR and only a brief transient activation of Erk1/2. In contrast, TGFα and EGF induced significant phosphorylation of EGFR and sustained activation of Erk1/2. While EGF-induced Erk phosphorylation gradually declined after 45 minutes, TGFα-stimulated cells maintained a steady level of phosphorylated Erk up to 180 minutes. Similarly, both EGF and TGFα induced phosphorylation of Akt which lasted up to 90 minutes, whereas AREG stimulated cells showed little Akt phosphorylation. Furthermore, the levels of total EGFR declined rapidly after EGF stimulation and almost no EGFR was detected 90 minutes after stimulation, whereas TGFα-stimulated cells maintained a considerable amount of EGFR at the same time point. EGFR level remained unchanged upon AREG stimulation.

### Differential effects of MEK and PI3K inhibition on differentiation of K5^+^K19^-^ hMEC cells

**Figure 5:**
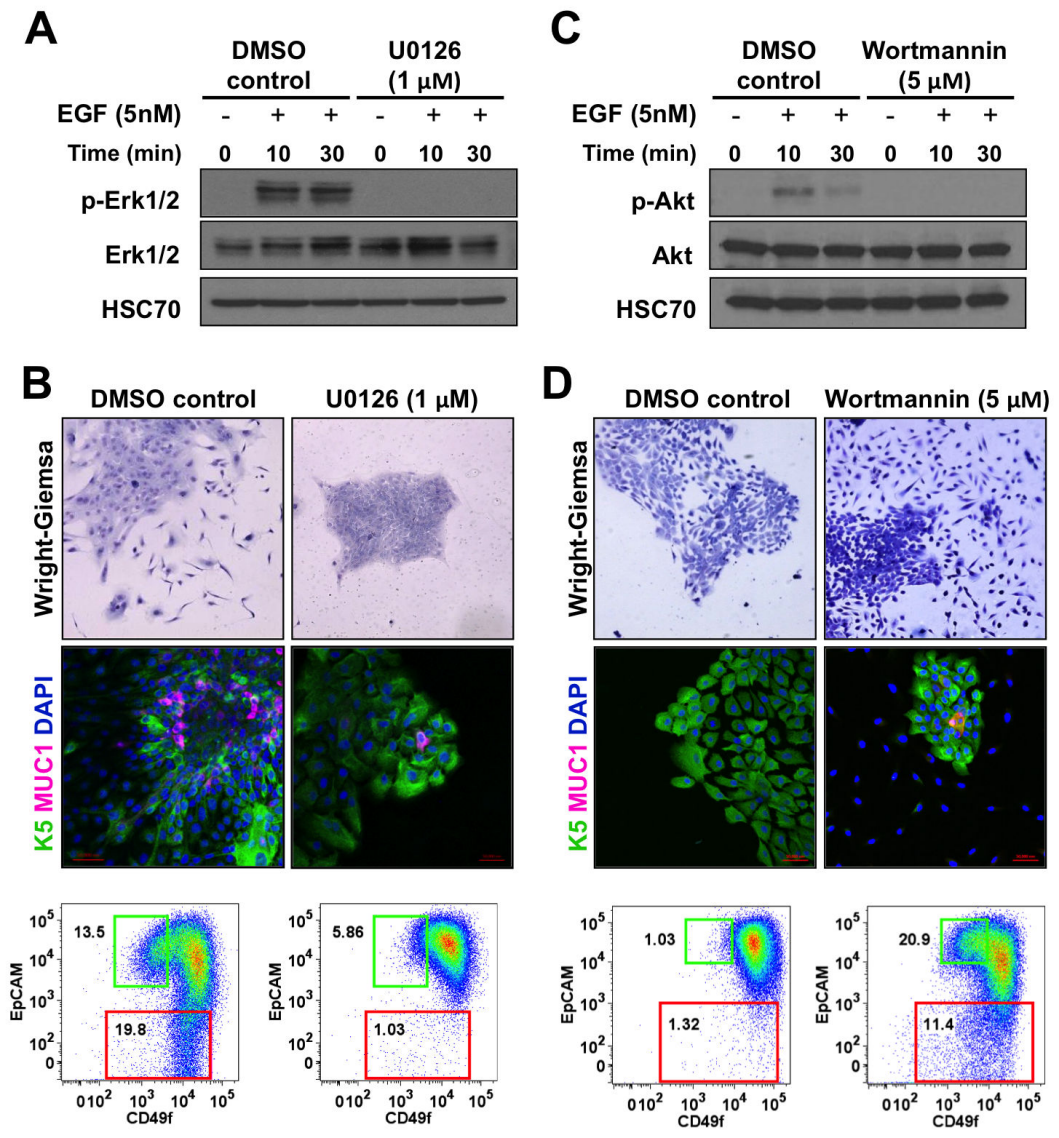
MEK inhibitor blocks differentiation of K5^+^K19^-^ hMECs. (**A**) Undifferentiated K5^+^K19^-^ hMECs were starved of serum/growth factors for 48 hours in D3 medium, treated with 1 µM U0126 or vehicle (DMSO) alone for 4 hours before stimulation with 5 nM EGF for the indicated period. Cell lysate was analyzed by immunoblotting. (**B**) K5^+^K19^-^ hMECs were propagated in MEGM medium (containing EGF) with or without 1 µM U0126 for three weeks. Medium was replaced every two days. Overall cell morphology was assessed by Wright-Giemsa staining (top panels) and K5 (green) and MUC1 (red) expression was assessed by immunofluorescence microscopy (middle panels). Nuclei were visualized with DAPI (blue). Red bars indicate 50 µM. Expression of CD49f and EpCAM was analyzed by flow cytometry (bottom panels). Gates and percentages for CD49f^lo^EpCAM^hi^ (luminal, green box) and EpCAM^lo^ (myoepithelial, red box) populations are indicated. Shown are representative results from 3 independent experiments. (**C**) Undifferentiated K5^+^K19^-^ hMECs were starved of serum/growth factors for 48 hours in D3 medium, treated with 5 µM wortmannin or vehicle (DMSO) alone for 4 hours before stimulation with 5 nM EGF for the indicated period. Cell lysate was analyzed by immunoblotting. (**D**) K5^+^K19^-^ hMECs were propagated in MEGM medium (containing EGF) with or without 5 µM wortmannin for ten days. At this time point, control culture has not differentiated yet. Medium was replaced every two days. Overall cell morphology was assessed by Wright-Giemsa staining (top panels) and K5 (green) and MUC1 (red) expression was assessed by immunofluorescence microscopy (middle panels). Nuclei were visualized with DAPI (blue). Red bars indicate 50 µM. Expression of CD49f and EpCAM was analyzed by flow cytometry (bottom panels). Gates and percentages for CD49f^lo^EpCAM^hi^ (luminal, green box) and EpCAM^lo^ (myoepithelial, red box) populations are indicated. Shown are representative results from 2 independent experiments.

The biochemical analyses showed that both Erk and Akt were activated more robustly upon acute stimulation with EGF and TGFα than by AREG. To examine whether either of these pathways can directly regulate K5^+^K19^-^ hMEC differentiation, we performed *in vitro* differentiation assays in the presence of chemical inhibitors of MEK and PI3K. First, we cultured K5^+^K19^-^ hMECs in MEGM medium (containing EGF) for three weeks with a MEK inhibitor U0126 and examined differentiation by fluorescence microscopy and flow cytometry. Treatment with U0126 did not affect cell growth at the concentration tested (Figure S5). As shown in Figure 5B, K5 ^+^ K19^-^ hMECs differentiated normally when treated with vehicle (DMSO) alone. However, cell differentiation was impaired in the presence of U0126. Specifically, significantly reduced number of spindle-shaped cells were observed around the tightly-packed epithelial clusters and essentially all cells maintained K5 expression. Flow cytometry confirmed that the percentages of both CD49f^lo^EpCAM^hi^ and EpCAM^lo^ cells were markedly reduced compared to vehicle-treated cells. The efficacy of U0126 was confirmed by probing for inhibition of Erk activation (Figure 5A).

In contrast, when K5^+^K19^-^ hMECs were cultured in the presence of a PI3K inhibitor wortmannin, the emergence of differentiated (CD49f^lo^EpCAM^hi^ and EpCAM^lo^) cells was accelerated. As shown in Figure 5D, 10 days after cells were placed in MEGM medium when vehicle-treated cells have not differentiated yet, wortmannin-treated cells already showed morphological changes of differentiation and flow cytometry profiles were consistent with these observations. We confirmed similar effects with another PI3K inhibitor LY294002 (Figure S6).

From these data, we concluded that Erk activation, but not Akt activation, downstream of EGFR is required for differentiation of K5^+^K19^-^ hMECs in MEGM medium.

## Discussion

Here we described an additional characterization of the hTERT-immortalized stem/progenitor K5^+^K19^-^ hMEC line *in vitro* differentiation model and its application to dissect biochemical mechanisms of MEC differentiation. By combining immunofluorescence microscopy, flow cytometry and biochemistry, we established that K5^+^K19^-^ hMECs, which express CD49f^hi^EpCAM^hi^ under non-differentiating condition, differentiated into CD49f^lo^EpCAM^hi^ and EpCAM^lo^ cells. In the culture conditions examined here, this process can be regulated by signals through EGFR as demonstrated by distinct effects of EGFR ligands EGF, AREG and TGFα in MEGM medium. Downstream of EGFR, Erk activation is required to promote differentiation, whereas Akt activation counters this process.

Though activation of both Erk [33] and Akt [34] have been strongly tied to breast cancer, our knowledge into their precise roles in mammary gland development is not complete. Roles of Erk on cell fate determination were first investigated in the PC12 neural cell line. In this system, a transient activation of Erk was associated with cell proliferation while a more prolonged phosphorylation of Erk led to cell differentiation [35,36]. This concept was later extended to the mammary morphogenesis and lineage determination. Using primary mouse MECs, Fata et al. demonstrated that TGFα induced sustained activation of Erk and promoted branching morphogenesis whereas transient Erk activation by FGF7 promoted cell growth without branching [37]. In a comparable study using human cells, Pasic et al. showed that transient Erk activation by AREG promoted emergence of both luminal and myoepithelial cells while sustained Erk activation by EGF favored myoepithelial differentiation [23].

In the present study, we showed that the AREG-containing MEGM medium promoted luminal differentiation of K5^+^K19^-^ hMECs while substitution with TGFα enhanced myoepithelial differentiation. Consistent with previous reports, stimulation with AREG induced transient Erk activation whereas more sustained activation was observed upon TGFα stimulation. Biochemical events from the acute phase of cell stimulation may not completely capture the continuously-unfolding changes in the complex signaling network over extended period required to induce cell differentiation. Nevertheless, effects of EGFR ligands on K5^+^K19^-^ hMEC differentiation appear to be dependent on the MEK-Erk axis because chemical inhibition of this pathway, but not the PI3K-Akt pathway, interfered with this process. In addition, luminal differentiation appears to be less sensitive to MEK inhibition because the percentage of CD49f^lo^EpCAM^hi^ (luminal) cells remained relatively unchanged while that of EpCAM^lo^ (myoepithelial) cells was reduced at lower concentrations of U0126 (Figure S4). Even at the highest concentration tested, a small number of MUC1^pos^ cells were repeatedly observed (Figure 5B). These results are essentially in agreement with findings by Pasic et al., which demonstrated that sustained activation of Erk through EGFR favored myoepithelial differentiation.

Nonetheless, some differences do exist in the spectrum of differentiated MECs between Pasic et al’s results and the present study. Specifically, in the former, AREG promoted luminal as well as myoepithelial differentiation whereas EGF favored only myoepithelial differentiation. These differences may be due to that Pasic et al. utilized primary human MECs, which are intrinsically more heterogeneous than K5^+^K19^-^ hMECs, which originated from a single clone [12]. Therefore, it is conceivable that their cell preparation contained progenitors capable of differentiating into both luminal and myoepithelial lineage upon AREG stimulation, whereas the K5^+^K19^-^ hMECs originated from a clone which can only produce luminal cells with AREG. Additional differences in culture conditions between these studies include the presence of extracellular matrix and the composition of the base medium. Though previous studies demonstrated that hTERT alone could not transform MECs [38,39], its effects on MEC differentiation have not been fully clarified. These questions need to be addressed in future studies.

The PI3K-Akt axis has been shown to exert diverse effects on the normal and pathological development of mammary glands. In mice, loss of Akt1 inhibited, but loss of another family member Akt2 accelerated mammary tumorigenesis [40]. Conditional activation of this pathway either through deletion of a negative regulator PTEN or over-expression of Akt induced precocious lactogenic differentiation of mammary epithelial cells [41,42]. These data apparently contradict our present findings that inhibition of the PI3K pathway accelerated differentiation of K5^+^K19^-^ hMECs.

However, activation of the PI3K-Akt pathway is also associated with the expansion of the mammary stem/progenitor populations and inhibition of differentiation [43]. Similar effects have been reported in other systems including embryonic stem cells [44] and neural stem cells [45]. One possible interpretation of these conflicting observations is that PIK3-Akt activity is required to maintain undifferentiated stem/progenitor state at the cell-autonomous level, but signals from tissue microenvironment can further modulate the ultimate outcome [46]. Collectively, our current results demonstrate that MEC differentiation is regulated by an intricate interplay between differentiation-promoting and -inhibiting signals, highlighting the complexity of the regulatory mechanisms of stem/progenitor cell maintenance and differentiation.

The biochemical and cell biological ramification of EGFR activation have been an object of extensive investigation. A number of studies demonstrated that AREG bound to EGFR with a much lower affinity than EGF, and while EGF-stimulated EGFR are internalized, ubiquitinated and degraded in the lysosome, receptor engagement with AREG or TGFα induced less degradation and more recycling [31,32]. Our preliminary investigation in K5^+^K19^-^ hMECs were consistent with published reports; a hundred (100) fold excess non-labeled AREG failed to displace fluorescence-labeled EGF, while excess non-labeled EGF or TGFα completely inhibited binding of labeled EGF (data not shown). All three ligands induced EGFR internalization at 5 nM within 10 minutes, but their intracellular fate differed significantly; EGFR trafficked to lysosomes and degraded upon EGF stimulation, whereas most EGFR recycled to the cell surface upon AREG stimulation (data not shown). In light of these observations, one possible interpretation of our present data is that the presence of low affinity EGFR ligand in MEGM medium promotes luminal differentiation and, as ligands bind with increasing affinity, cells begin to differentiate into myoepithelial lineage. This prompted us to examine whether lower concentrations of high affinity ligands mimic biological activity of low affinity ligands. However, when we cultured K5^+^K19^-^ hMECs in MEGM media containing varying concentrations of EGFR ligands, presence of lower concentrations of TGFα or EGF did not increase MUC1^pos^ cells, indicating that luminal differentiation was not augmented (Figure S2). Furthermore, though both AREG and TGFα are known to promote EGFR recycling rather than degradation, these ligands in MEGM medium showed opposing effects on K5^+^K19^-^ hMEC differentiation, AREG favoring luminal lineage and TGFα myoepithelial lineage. Altogether, present results indicate that biological consequences of EGFR engagement are not dictated by single factors such as receptor occupancy, binding affinity or receptor trafficking, but likely to be governed by the interaction of multiple determinants.

In conclusion, the present data validate the utility of the K5^+^K19^-^ hMEC cells for modeling key features of human MEC differentiation. We found that different EGFR ligands within MEGM medium could promote preferential differentiation into either luminal or myoepithelial fate. These findings open ways to dissect precise molecular/biochemical mechanisms of MEC differentiation, and we envision the K5^+^K19^-^ hMEC cells to be a useful model for this purpose.

## Supporting Information

Figure S1 **Expression of K5 after differentiation.** Cells were either maintained under non-differentiating condition (DFCI-1 medium) or propagated under differentiation-promoting condition (MEGM medium containing 5 nM EGF) for three weeks and expression of CD49f, EpCAM and K5 was assessed by flow cytometry. Cells were fixed and permeabilized for intracellular K5 staining. Gates for CD49f^lo^EpCAM^hi^ (luminal, green box), EpCAM^lo^ (myoepithelial, red box) and CD49f^hi^EpCAM^hi^ (undifferentiated, black box) cells are indicated. Note that CD49f and EpCAM expression patterns are slightly altered compared to those in Figures 1, 2 and 5 due to cell fixation and permeabilization. Histograms indicate levels of K5 in cells propagated in MEGM medium. The green line represents the levels of K5 in the CD49f^lo^EpCAM^hi^ (luminal) population, the red line is for the EpCAM^lo^ (myoepithelial) population and the black line for the CD49f^hi^EpCAM^hi^ (undifferentiated) population. Mean fluorescence intensity of K5 for CD49f^lo^EpCAM^hi^ (luminal), EpCAM^lo^ (myoepithelial) and CD49f^hi^EpCAM^hi^ (undifferentiated) populations are 1802, 806 and 1695, respectively.
(TIF)

Figure S2 **Effect of varying doses of EGFR ligands in MEGM medium on MEC differentiation.** K5^+^K19^-^ hMECs were propagated in modified MEGM medium containing indicated concentrations of EGFR ligands EGF, AREG or TGFα for three weeks. Cell differentiation was evaluated by K5 (green) and MUC1 (purple) staining. Nuclei were visualized with DAPI (blue). Red bars indicate 50 µM.
(TIF)

Figure S3 **Cell morphology after sort.** K5^+^K19^-^ hMECs were propagated in MEGM medium (containing EGF) for three weeks and sorted based on CD49f and EpCAM expression. Sorted CD49f^lo^EpCAM^hi^ (luminal) and EpCAM^lo^ (myoepithelial) populations cells were seeded into modified MEGM medium where EGF was substituted with AREG or TGFα. Cell morphology was documented three days later.
(TIF)

Figure S4 **Effect of varying doses of MEK inhibitor on differentiation.** K5^+^K19^-^ hMECs were propagated in MEGM medium (containing EGF) with indicated concentrations of U0126 for three weeks. Medium was replaced every two days. Expression of CD49f and EpCAM was analyzed by flow cytometry. Gates and percentages for CD49f^lo^EpCAM^hi^ (luminal, green box) and EpCAM^lo^ (myoepithelial, red box) populations are indicated.
(TIF)

Figure S5 **Effect of U0126 and wortmannin on cell growth.** K5^+^K19^-^ hMECs were seeded in MEGM medium (with 5 nM EGF) in 6 well plates at 10^4^ cells/well and effects of U0126 and wortmannin on cell growth were evaluated. Cells were detached from plates at indicated time points and live cell numbers were determined. Shown are average cell numbers from 6 replicates. Error bars indicate standard errors. There was no statistically significant difference between DMSO and U0126 treatment groups; Wortmannin treatment significantly inhibited cell growth.
(TIF)

Figure S6 **Effect of LY294002 on differentiation.** K5^+^K19^-^ hMECs were cultured in MEGM medium (containing EGF) for 8 days in the presence or absence of 0.5 µM LY294002 and cell differentiation was evaluated by flow cytometry.
(TIF)

## References

[1] Perou CM, Sørlie T, Eisen MB, Van de Rijn M, Jeffrey SS et al. (2000) Molecular portraits of human breast tumours. Nature 406: 747–752. doi:10.1038/35021093. PubMed: 10963602. 10963602

[2] Sørlie T, Perou CM, Tibshirani R, Aas T, Geisler S et al. (2001) Gene expression patterns of breast carcinomas distinguish tumor subclasses with clinical implications. Proc Natl Acad Sci U S A 98: 10869–10874. doi:10.1073/pnas.191367098. PubMed: 11553815. 11553815 PMC58566

[3] Sorlie T, Tibshirani R, Parker J, Hastie T, Marron JS et al. (2003) Repeated observation of breast tumor subtypes in independent gene expression data sets. Proc Natl Acad Sci U S A 100: 8418–8423. doi:10.1073/pnas.0932692100. PubMed: 12829800. 12829800 PMC166244

[4] Van Keymeulen A, Rocha AS, Ousset M, Beck B, Bouvencourt G et al. (2011) Distinct stem cells contribute to mammary gland development and maintenance. Nature 479: 189–193. doi:10.1038/nature10573. PubMed: 21983963. 21983963

[5] Van Amerongen R, Bowman AN, Nusse R (2012) Developmental stage and time dictate the fate of Wnt/β-catenin-responsive stem cells in the mammary gland. Cell Stem Cell 11: 387–400. doi:10.1016/j.stem.2012.05.023. PubMed: 22863533. 22863533 PMC13155203

[6] De Visser KE, Ciampricotti M, Michalak EM, Tan DW-M, Speksnijder EN et al. (2012) Developmental stage-specific contribution of LGR5(+) cells to basal and luminal epithelial lineages in the postnatal mammary gland. J Pathol 228: 300–309. doi:10.1002/path.4096. PubMed: 22926799. 22926799

[7] Lim E, Vaillant F, Wu D, Forrest NC, Pal B et al. (2009) Aberrant luminal progenitors as the candidate target population for basal tumor development in BRCA1 mutation carriers. Nat Med 15: 907–913. doi:10.1038/nm.2000. PubMed: 19648928. 19648928

[8] Proia TA, Keller PJ, Gupta PB, Klebba I, Jones AD et al. (2011) Genetic predisposition directs breast cancer phenotype by dictating progenitor cell fate. Cell Stem Cell 8: 149–163. doi:10.1016/j.stem.2010.12.007. PubMed: 21295272. 21295272 PMC3050563

[9] Molyneux G, Geyer FC, Magnay F-A, McCarthy A, Kendrick H et al. (2010) BRCA1 basal-like breast cancers originate from luminal epithelial progenitors and not from basal stem cells. Cell Stem Cell 7: 403–417. doi:10.1016/j.stem.2010.07.010. PubMed: 20804975. 20804975

[10] Guo W, Keckesova Z, Donaher JL, Shibue T, Tischler V et al. (2012) Slug and Sox9 Cooperatively Determine the Mammary Stem Cell State. Cell 148: 1015–1028. doi:10.1016/j.cell.2012.02.008. PubMed: 22385965. 22385965 PMC3305806

[11] Hennighausen L, Robinson GW (2005) Information networks in the mammary gland. Nat Rev Mol Cell Biol 6: 715–725. doi:10.1038/nrm1714. PubMed: 16231422. 16231422

[12] Zhao X, Malhotra GK, Lele SM, Lele MS, West WW et al. (2010) Telomerase-immortalized human mammary stem/progenitor cells with ability to self-renew and differentiate. Proc Natl Acad Sci U S A 107: 14146–14151. doi:10.1073/pnas.1009030107. PubMed: 20660721. 20660721 PMC2922525

[13] Sternlicht MD, Sunnarborg SW (2008) The ADAM17-amphiregulin-EGFR axis in mammary development and cancer. J Mammary Gland Biol Neoplasia 13: 181–194. doi:10.1007/s10911-008-9084-6. PubMed: 18470483. 18470483 PMC2723838

[14] Fowler KJ, Walker F, Alexander W, Hibbs ML, Nice EC et al. (1995) A mutation in the epidermal growth factor receptor in waved-2 mice has a profound effect on receptor biochemistry that results in impaired lactation. Proc Natl Acad Sci U S A 92: 1465–1469. doi:10.1073/pnas.92.5.1465. PubMed: 7533293. 7533293 PMC42540

[15] Luetteke NC, Qiu TH, Fenton SE, Troyer KL, Riedel RF et al. (1999) Targeted inactivation of the EGF and amphiregulin genes reveals distinct roles for EGF receptor ligands in mouse mammary gland development. Development 126: 2739–2750. PubMed: 10331984. 10331984 10.1242/dev.126.12.2739

[16] Nielsen TO, Hsu FD, Jensen K, Cheang M, Karaca G et al. (2004) Immunohistochemical and clinical characterization of the basal-like subtype of invasive breast carcinoma. Clin Cancer Res 10: 5367–5374. doi:10.1158/1078-0432.CCR-04-0220. PubMed: 15328174. 15328174

[17] Livasy CA, Karaca G, Nanda R, Tretiakova MS, Olopade OI et al. (2006) Phenotypic evaluation of the basal-like subtype of invasive breast carcinoma. Mod Pathol 19: 264–271. doi:10.1038/modpathol.3800528. PubMed: 16341146. 16341146

[18] Derynck R, Goeddel DV, Ullrich A, Gutterman JU, Williams RD et al. (1987) Synthesis of messenger RNAs for transforming growth factors alpha and beta and the epidermal growth factor receptor by human tumors. Cancer Res 47: 707–712. PubMed: 3467839. 3467839

[19] Bates SE, Davidson NE, Valverius EM, Freter CE, Dickson RB et al. (1988) Expression of transforming growth factor alpha and its messenger ribonucleic acid in human breast cancer: its regulation by estrogen and its possible functional significance. Mol Endocrinol 2: 543–555. doi:10.1210/mend-2-6-543. PubMed: 3047554. 3047554

[20] Dotzlaw H, Miller T, Karvelas J, Murphy LC (1990) Epidermal growth factor gene expression in human breast cancer biopsy samples: relationship to estrogen and progesterone receptor gene expression. Cancer Res 50: 4204–4208. PubMed: 2364377. 2364377

[21] LeJeune S, Leek R, Horak E, Plowman G, Greenall M et al. (1993) Amphiregulin, epidermal growth factor receptor, and estrogen receptor expression in human primary breast cancer. Cancer Res 53: 3597–3602. PubMed: 8101763. 8101763

[22] Qi CF, Liscia DS, Normanno N, Merlo G, Johnson GR et al. (1994) Expression of transforming growth factor alpha, amphiregulin and cripto-1 in human breast carcinomas. Br J Cancer 69: 903–910. doi:10.1038/bjc.1994.174. PubMed: 8180021. 8180021 PMC1968887

[23] Pasic L, Eisinger-Mathason TSK, Velayudhan BT, Moskaluk CA, Brenin DR et al. (2011) Sustained activation of the HER1-ERK1/2-RSK signaling pathway controls myoepithelial cell fate in human mammary tissue. Genes Dev 25: 1641–1653. doi:10.1101/gad.2025611. PubMed: 21828273. 21828273 PMC3182019

[24] Band V, Sager R (1989) Distinctive traits of normal and tumor-derived human mammary epithelial cells expressed in a medium that supports long-term growth of both cell types. Proc Natl Acad Sci U S A 86: 1249–1253. doi:10.1073/pnas.86.4.1249. PubMed: 2919173. 2919173 PMC286665

[25] Band V, Zajchowski D, Kulesa V, Sager R (1990) Human papilloma virus DNAs immortalize normal human mammary epithelial cells and reduce their growth factor requirements. Proc Natl Acad Sci U S A 87: 463–467. doi:10.1073/pnas.87.1.463. PubMed: 2153303. 2153303 PMC53284

[26] Maroni D, Davis JS (2011) TGFB1 disrupts the angiogenic potential of microvascular endothelial cells of the corpus luteum. J Cell Sci 124: 2501–2510. doi:10.1242/jcs.084558. PubMed: 21693577. 21693577 PMC6518331

[27] Stingl J, Eaves CJ, Zandieh I, Emerman JT (2001) Characterization of bipotent mammary epithelial progenitor cells in normal adult human breast tissue. Breast Cancer Res Treat 67: 93–109. doi:10.1023/A:1010615124301. PubMed: 11519870. 11519870

[28] Clayton H, Titley I, Vivanco Md dM (2004) Growth and differentiation of progenitor/stem cells derived from the human mammary gland. Exp Cell Res 297: 444–460. doi:10.1016/j.yexcr.2004.03.029. PubMed: 15212947. 15212947

[29] Stingl J, Raouf A, Eirew P, Eaves CJ (2006) Deciphering the mammary epithelial cell hierarchy. Cell Cycle 5: 1519–1522. doi:10.4161/cc.5.14.2983. PubMed: 16861925. 16861925

[30] Villadsen R, Fridriksdottir AJ, Rønnov-Jessen L, Gudjonsson T, Rank F et al. (2007) Evidence for a stem cell hierarchy in the adult human breast. J Cell Biol 177: 87–101. doi:10.1083/jcb.200611114. PubMed: 17420292. 17420292 PMC2064114

[31] Roepstorff K, Grandal MV, Henriksen L, Knudsen SLJ, Lerdrup M et al. (2009) Differential effects of EGFR ligands on endocytic sorting of the receptor. Traffic 10: 1115–1127. doi:10.1111/j.1600-0854.2009.00943.x. PubMed: 19531065. 19531065 PMC2723868

[32] Baldys A, Göoz M, Morinelli TA, Lee M-H, Raymond JR et al. (2009) Essential role of c-Cbl in amphiregulin-induced recycling and signaling of the endogenous epidermal growth factor receptor. Biochemistry 48: 1462–1473. doi:10.1021/bi801771g. PubMed: 19173594. 19173594 PMC2645952

[33] Whyte J, Bergin O, Bianchi A, McNally S, Martin F (2009) Key signalling nodes in mammary gland development and cancer. Mitogen-activated protein kinase signalling in experimental models of breast cancer progression and in mammary gland development. Breast Cancer Res 11: 209. doi:10.1186/bcr2361. PubMed: 19818165. 19818165 PMC2790844

[34] Wickenden JA, Watson CJ (2010) Key signalling nodes in mammary gland development and cancer. Signalling downstream of PI3 kinase in mammary epithelium: a play in 3 Akts. Breast Cancer Res 12: 202. doi:10.1186/bcr2558. PubMed: 20398329. 20398329 PMC2879565

[35] Qui MS, Green SH (1992) PC12 cell neuronal differentiation is associated with prolonged p21ras activity and consequent prolonged ERK activity. Neuron 9: 705–717. doi:10.1016/0896-6273(92)90033-A. PubMed: 1382473. 1382473

[36] Traverse S, Gomez N, Paterson H, Marshall C, Cohen P (1992) Sustained activation of the mitogen-activated protein (MAP) kinase cascade may be required for differentiation of PC12 cells. Comparison of the effects of nerve growth factor and epidermal growth factor. Biochem J 288(2: 351–355. 1334404 10.1042/bj2880351 PMC1132018

[37] Fata JE, Mori H, Ewald AJ, Zhang H, Yao E et al. (2007) The MAPK(ERK-1,2) pathway integrates distinct and antagonistic signals from TGFalpha and FGF7 in morphogenesis of mouse mammary epithelium. Dev Biol 306: 193–207. doi:10.1016/j.ydbio.2007.03.013. PubMed: 17448457. 17448457 PMC2763137

[38] Hahn WC, Counter CM, Lundberg AS, Beijersbergen RL, Brooks MW et al. (1999) Creation of human tumour cells with defined genetic elements. Nature 400: 464–468. doi:10.1038/22780. PubMed: 10440377. 10440377

[39] Elenbaas B (2001) Human breast cancer cells generated by oncogenic transformation of primary mammary epithelial cells. Genes Dev 15: 50–65. doi:10.1101/gad.828901. PubMed: 11156605. 11156605 PMC312602

[40] Maroulakou IG, Oemler W, Naber SP, Tsichlis PN (2007) Akt1 ablation inhibits, whereas Akt2 ablation accelerates, the development of mammary adenocarcinomas in mouse mammary tumor virus (MMTV)-ErbB2/neu and MMTV-polyoma middle T transgenic mice. Cancer Res 67: 167–177. doi:10.1158/0008-5472.CAN-06-3782. PubMed: 17210696. 17210696

[41] Li G, Robinson GW, Lesche R, Martinez-Diaz H, Jiang Z et al. (2002) Conditional loss of PTEN leads to precocious development and neoplasia in the mammary gland. Development 129: 4159–4170. PubMed: 12163417. 12163417 10.1242/dev.129.17.4159

[42] Chen C-C, Stairs DB, Boxer RB, Belka GK, Horseman ND et al. (2012) Autocrine prolactin induced by the Pten-Akt pathway is required for lactation initiation and provides a direct link between the Akt and Stat5 pathways. Genes Dev 26: 2154–2168. doi:10.1101/gad.197343.112. PubMed: 23028142. 23028142 PMC3465737

[43] Korkaya H, Paulson A, Charafe-Jauffret E, Ginestier C, Brown M et al. (2009) Regulation of Mammary Stem/Progenitor Cells by PTEN/Akt/β-Catenin Signaling. PLOS Biol 7: e1000121. doi:10.1371/journal.pbio.1000121. 19492080 PMC2683567

[44] Watanabe S, Umehara H, Murayama K, Okabe M, Kimura T et al. (2006) Activation of Akt signaling is sufficient to maintain pluripotency in mouse and primate embryonic stem cells. Oncogene 25: 2697–2707. doi:10.1038/sj.onc.1209307. PubMed: 16407845. 16407845

[45] Groszer M, Erickson R, Scripture-Adams DD, Lesche R, Trumpp A et al. (2001) Negative regulation of neural stem/progenitor cell proliferation by the Pten tumor suppressor gene in vivo. Science 294: 2186–2189. doi:10.1126/science.1065518. PubMed: 11691952. 11691952

[46] Venere M, Lathia JD, Rich JN (2013) Growth Factor Receptors Define Cancer Hierarchies. Cancer Cell 23: 135–137. doi:10.1016/j.ccr.2013.01.020. PubMed: 23410969. 23410969 PMC3616317

