# Supplementary figures and images for: Distinct Effects of EGFR Ligands on Human Mammary Epithelial Cell Differentiation
Source: PLoS One. 2013 Oct 4;8(10):e75907. doi: 10.1371/journal.pone.0075907 (PMC3790811; doi:10.1371/journal.pone.0075907)

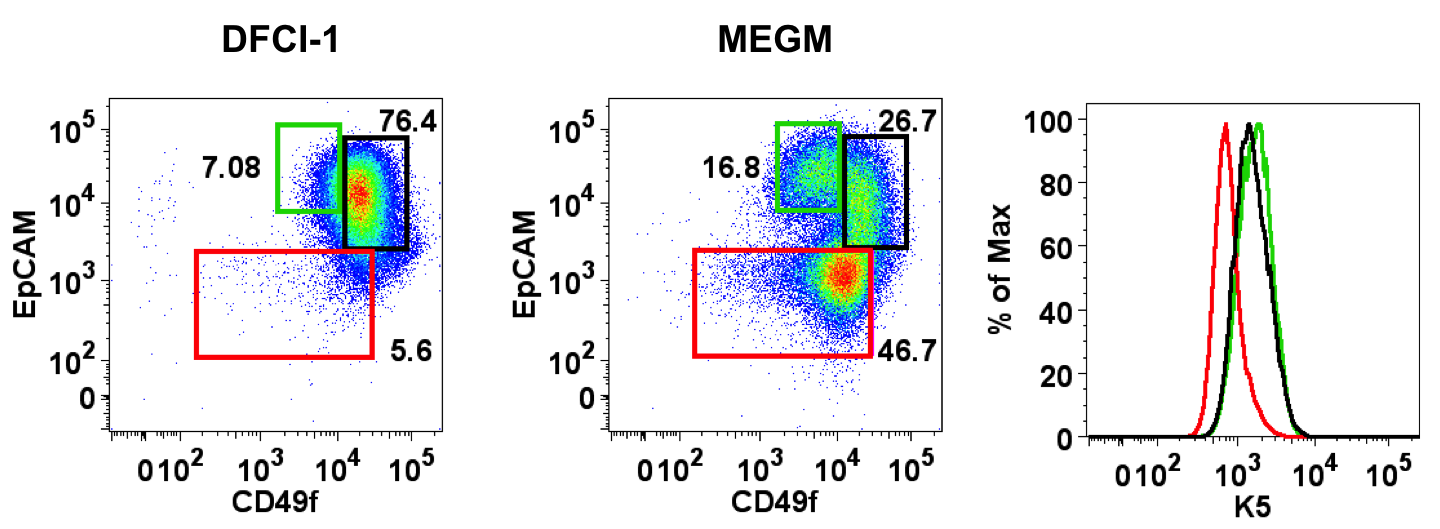

Supplement: Figure S1 — Expression of K5 after differentiation. Cells were either maintained under non-differentiating condition (DFCI-1 medium) or propagated under differentiation-promoting condition (MEGM medium containing 5 nM EGF) for three weeks and expression of CD49f, EpCAM and K5 was assessed by flow cytometry. Cells were fixed and permeabilized for intracellular K5 staining. Gates for CD49floEpCAMhi (luminal, green box), EpCAMlo (myoepithelial, red box) and CD49fhiEpCAMhi (undifferentiated, black box) cells are indicated. Note that CD49f and EpCAM expression patterns are slightly altered compared to those in Figures 1, 2 and 5 due to cell fixation and permeabilization. Histograms indicate levels of K5 in cells propagated in MEGM medium. The green line represents the levels of K5 in the CD49floEpCAMhi (luminal) population, the red line is for the EpCAMlo (myoepithelial) population and the black line for the CD49fhiEpCAMhi (undifferentiated) population. Mean fluorescence intensity of K5 for CD49floEpCAMhi (luminal), EpCAMlo (myoepithelial) and CD49fhiEpCAMhi (undifferentiated) populations are 1802, 806 and 1695, respectively. (TIF) [file pone.0075907.s001.tif]

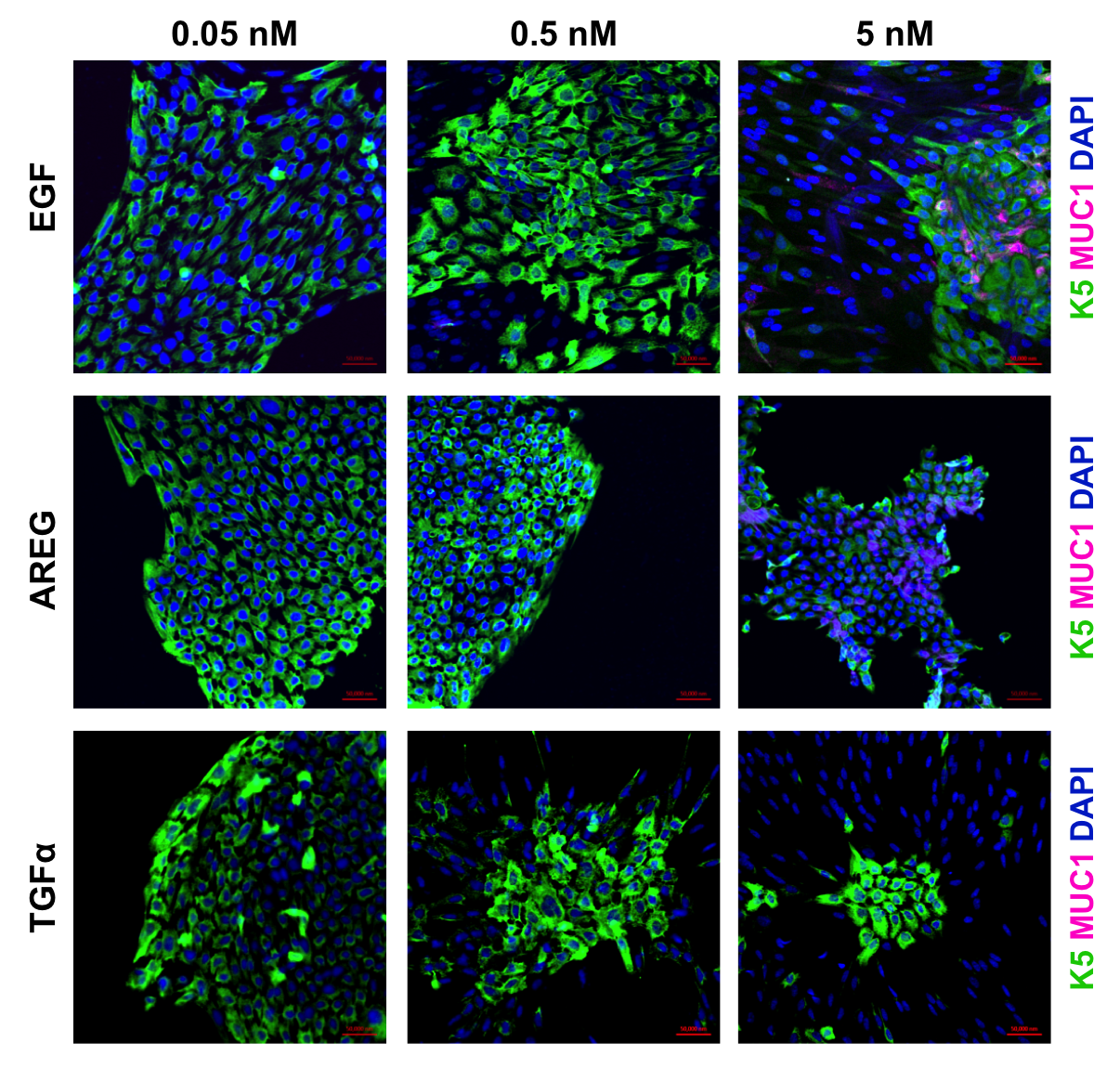

Supplement: Figure S2 — Effect of varying doses of EGFR ligands in MEGM medium on MEC differentiation. K5+K19- hMECs were propagated in modified MEGM medium containing indicated concentrations of EGFR ligands EGF, AREG or TGFα for three weeks. Cell differentiation was evaluated by K5 (green) and MUC1 (purple) staining. Nuclei were visualized with DAPI (blue). Red bars indicate 50 µM. (TIF) [file pone.0075907.s002.tif]

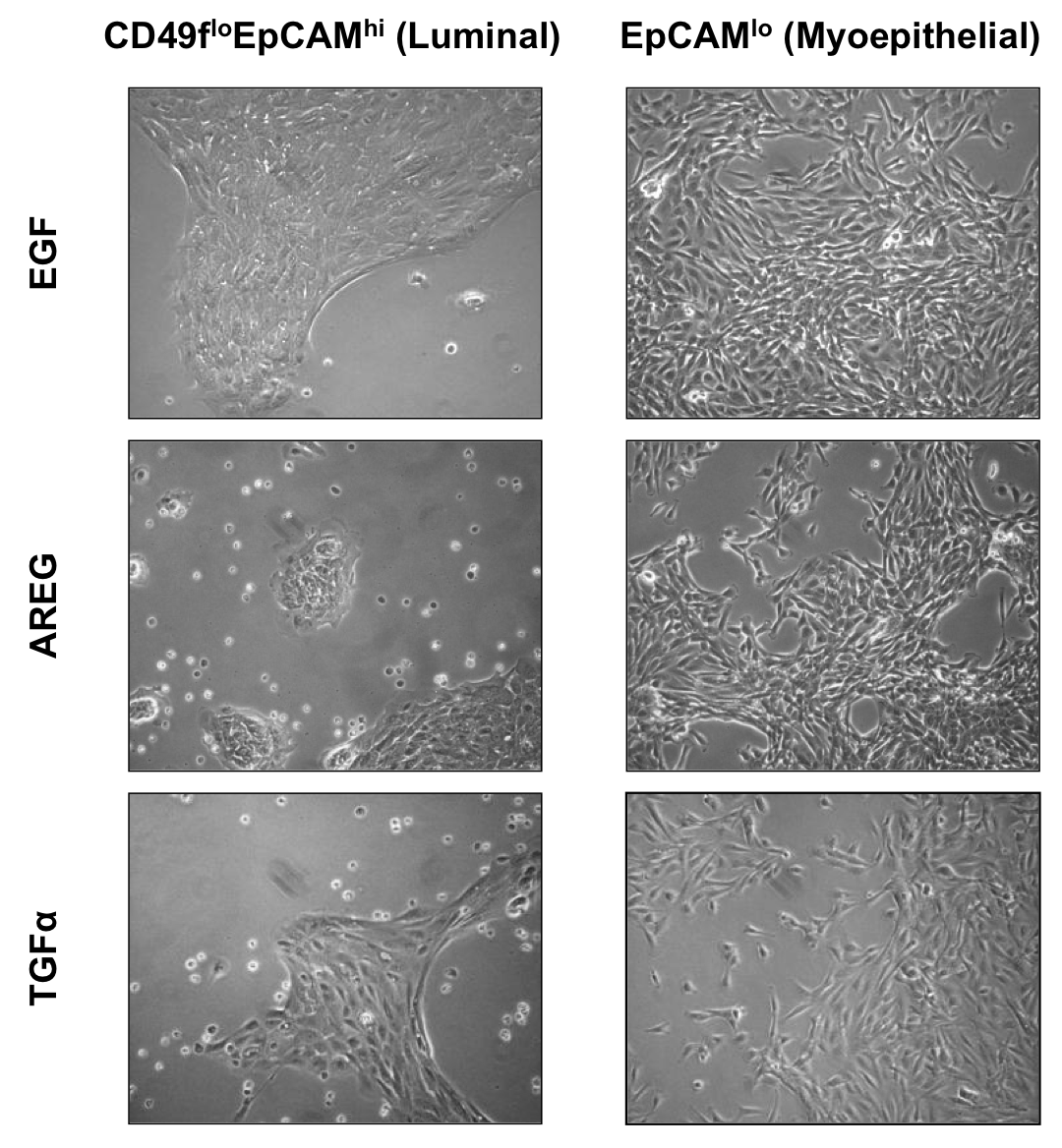

Supplement: Figure S3 — Cell morphology after sort. K5+K19- hMECs were propagated in MEGM medium (containing EGF) for three weeks and sorted based on CD49f and EpCAM expression. Sorted CD49floEpCAMhi (luminal) and EpCAMlo (myoepithelial) populations cells were seeded into modified MEGM medium where EGF was substituted with AREG or TGFα. Cell morphology was documented three days later. (TIF) [file pone.0075907.s003.tif]

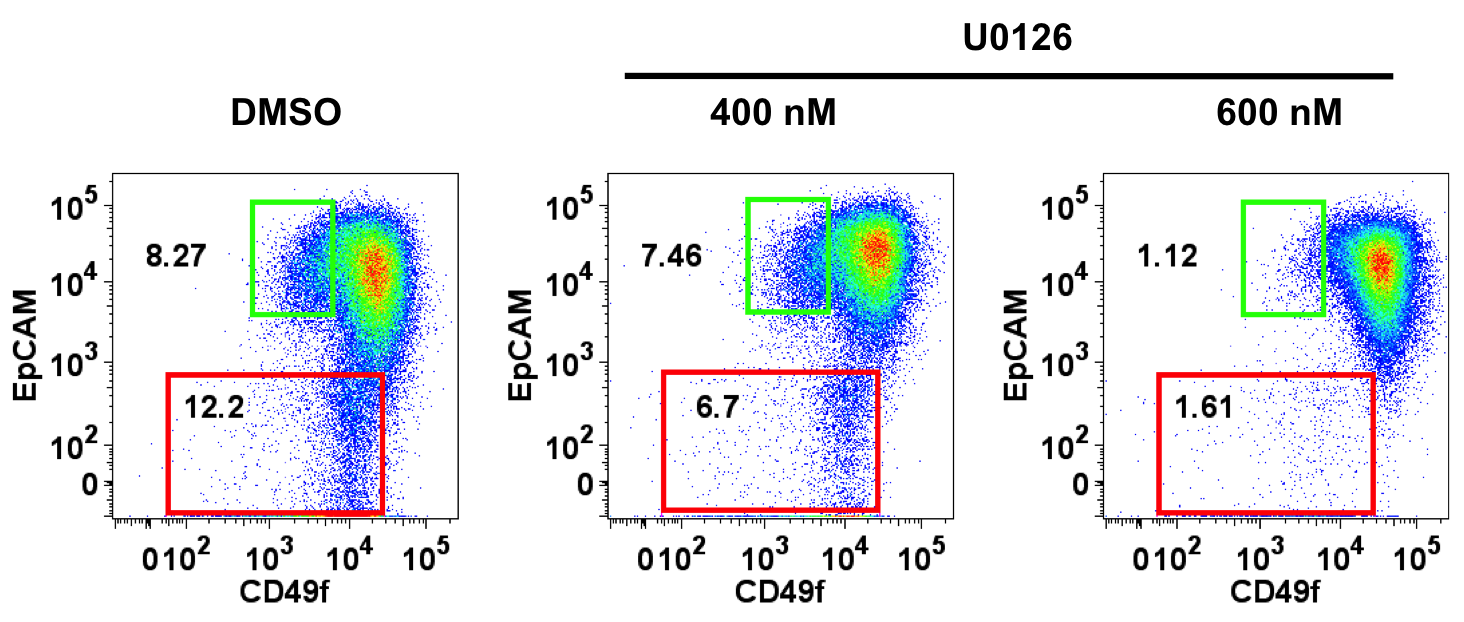

Supplement: Figure S4 — Effect of varying doses of MEK inhibitor on differentiation. K5+K19- hMECs were propagated in MEGM medium (containing EGF) with indicated concentrations of U0126 for three weeks. Medium was replaced every two days. Expression of CD49f and EpCAM was analyzed by flow cytometry. Gates and percentages for CD49floEpCAMhi (luminal, green box) and EpCAMlo (myoepithelial, red box) populations are indicated. (TIF) [file pone.0075907.s004.tif]

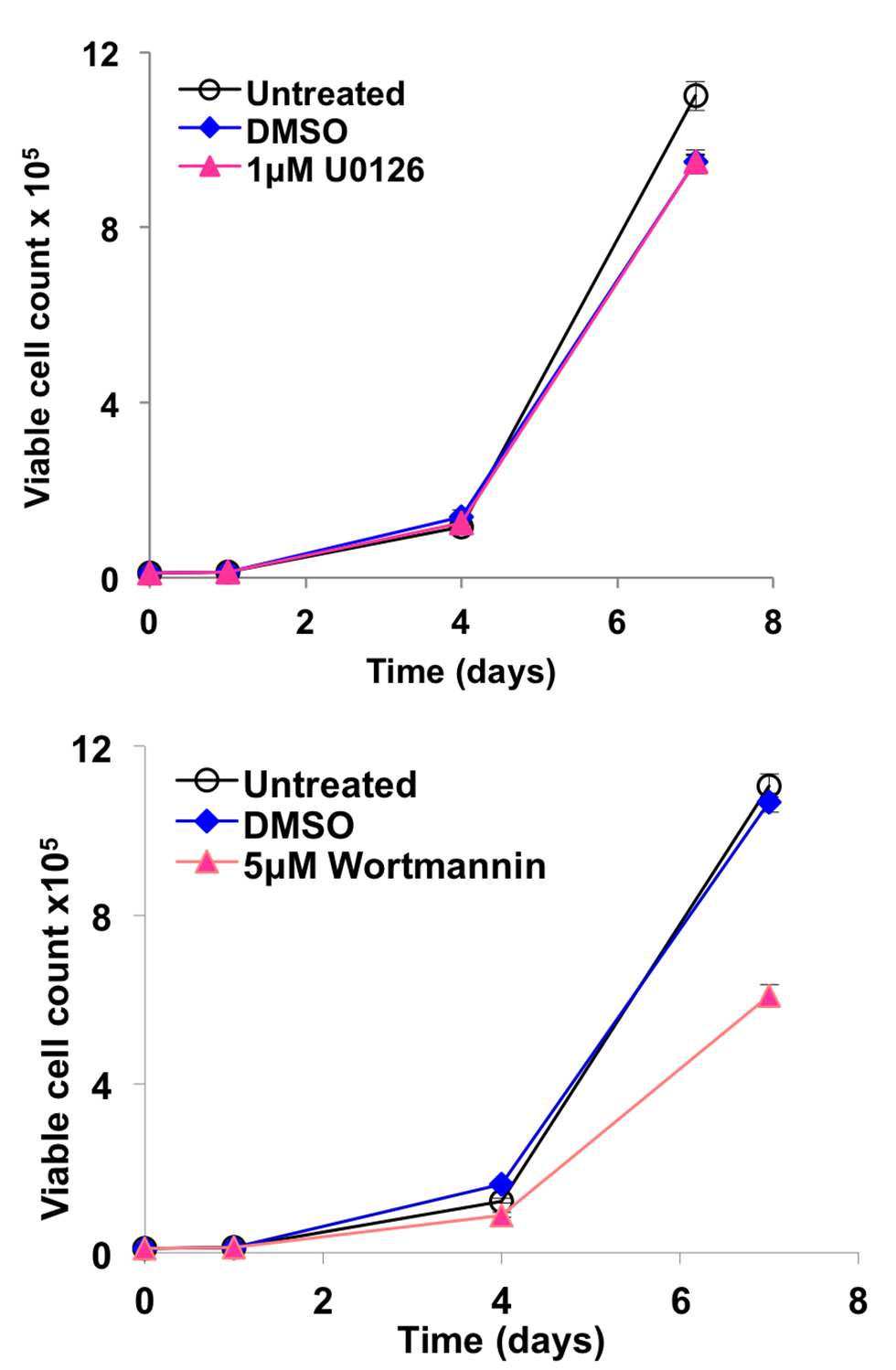

Supplement: Figure S5 — Effect of U0126 and wortmannin on cell growth. K5+K19- hMECs were seeded in MEGM medium (with 5 nM EGF) in 6 well plates at 104 cells/well and effects of U0126 and wortmannin on cell growth were evaluated. Cells were detached from plates at indicated time points and live cell numbers were determined. Shown are average cell numbers from 6 replicates. Error bars indicate standard errors. There was no statistically significant difference between DMSO and U0126 treatment groups; Wortmannin treatment significantly inhibited cell growth. (TIF) [file pone.0075907.s005.tif]

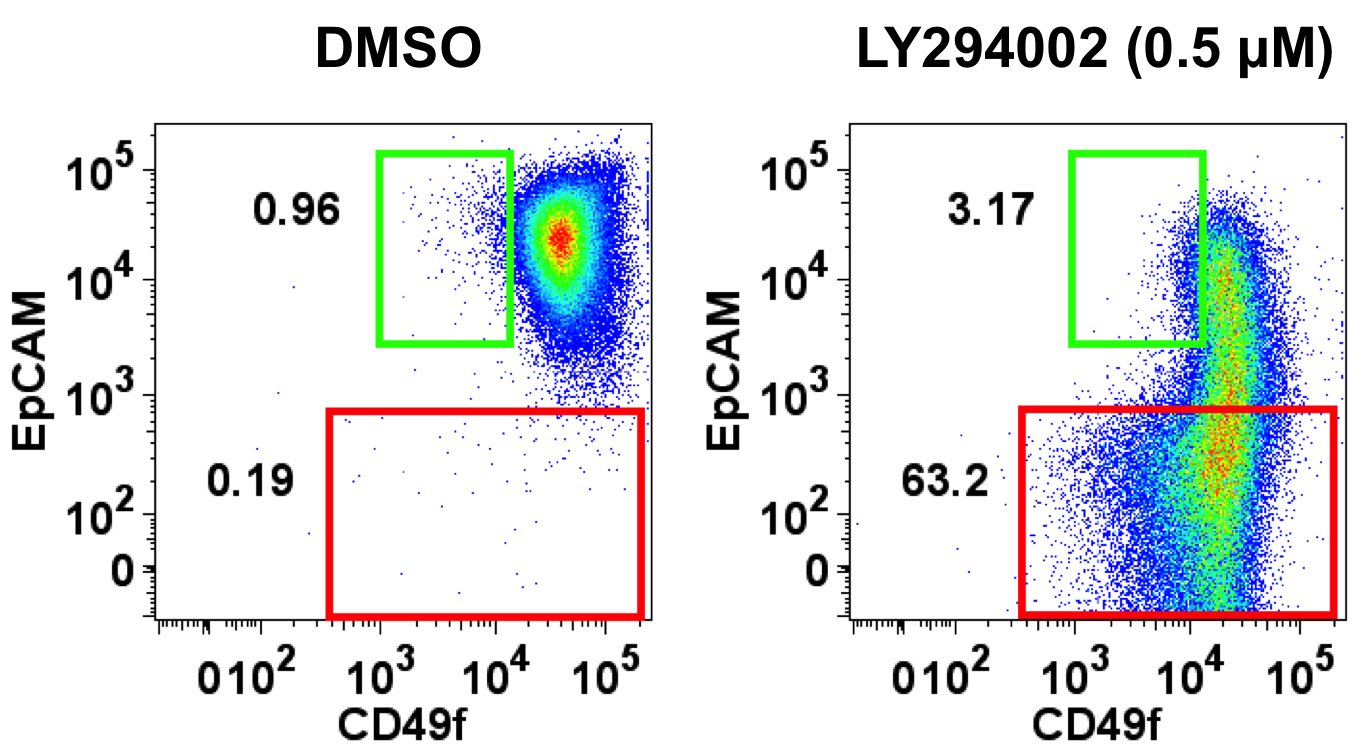

Supplement: Figure S6 — Effect of LY294002 on differentiation. K5+K19- hMECs were cultured in MEGM medium (containing EGF) for 8 days in the presence or absence of 0.5 µM LY294002 and cell differentiation was evaluated by flow cytometry. (TIF) [file pone.0075907.s006.tif]
